# To Be or Not to Be… An Antioxidant? That Is the Question

**DOI:** 10.3390/antiox9121234

**Published:** 2020-12-05

**Authors:** José M. Palma, Isabel Seiquer

**Affiliations:** 1 Department of Biochemistry, Cell and Molecular Biology of Plants, Estación Experimental del Zaidín, CSIC, 18008 Granada, Spain; 2 Department of Physiology and Biochemistry of Animal Nutrition, Estación Experimental del Zaidín, CSIC, Armilla, 18100 Granada, Spain

The concept of antioxidants refers to a substance with the capacity to either directly scavenge or indirectly prevent the formation of pro-oxidant molecules, basically associated to the so called reactive oxygen species (ROS). Considering the cell/tissue target, the picture is quite different. Thus, in animal tissues, the main source for ROS production is the mitochondria, whereas in plants, chloroplast is the most important organelle to generate ROS, including singlet oxygen (^1^O_2_), superoxide radicals (O_2_^●−^) or hydrogen peroxide (H_2_O_2_). Accordingly, and due to the respective peculiarities, each organelle/cell/tissue has its own antioxidant machinery to overcome the deleterious effects of their internal ROS levels and production. 

The real situation is that our cells are continually threatened by ROS, that disturb their normal and pacific living. Oxidative stress arises from an augmented ROS generation, but also from a decay of the antioxidant defense system, leading to the imbalance between the occurrence of reactive species and the organism’s ability to counteract them. Oxidative stress is responsible for cell damage, sometimes irrecoverable, which is further implicated in a cascade of degenerative diseases, cardiovascular diseases, cancer and aging. Therefore, in this dangerous situation, the endogenous defense system (which includes antioxidant enzymes and non-enzymatic compounds such as glutathione, vitamins, coenzyme Q and others) needs external help to lower/modulate the negative effects of excessive ROS. This exogenous help is represented by the dietary antioxidants, i.e., food antioxidants, most of them present in fruits and vegetables or other sources from the plant kingdom.

Oxidative stress is also suffered by plants, in which illumination conditions and excess of light may lead to ROS formation. Chloroplasts contain a well-equipped battery of both enzymatic and non-enzymatic antioxidants to reestablish the initial balanced state and to avoid a situation of oxidative stress. In this response mainly participate the molecular antioxidants α-tocopherol, carotenoids and ascorbate, the enzyme superoxide dismutase and the ascorbate glutathione cycle (AGC) which involves ascorbate, glutathione, NADPH and four redox enzymes. Some of these antioxidants, which in plants are required in great amounts to cope against the stressful conditions, have vitamin properties and may function as dietary antioxidants for animal and human beings. Thus, α-tocopherol is the chemical nature of vitamin E, whereas β-carotene is precursor of vitamin A once the plant carotene is assimilated by our metabolism; ascorbate is vitamin C, one of the most powerful antioxidants and has the ability to directly scavenge most ROS. Besides this antioxidant team, plants contain a huge amount of secondary metabolites with potential antioxidant activity.

As indicated above, the antioxidants are usually required in high quantities to neutralize the oxidative effects of ROS in plants. This is in contradiction with the concept of vitamins in the animal/human scenario, which is considered as an organic molecule that is essential in small quantities for the proper functioning of the organism’s metabolism. Commonly, in animals and humans these essential micronutrients are insufficiently or not synthesized and they need to obtain them through the diet. Whereas in plant systems, α-tocopherol and carotenoids are basically used as singlet oxygen scavengers, thus protecting cells against this ROS, in animal cells vitamins A and E play important roles regulating the redox homeostasis in a series of physiological processes. Thus, vitamin A is crucial for vision, growth and development, and for protecting epithelium and mucus integrity in the respiratory, urinary and gastro intestinal tract. Likewise, vitamin E seems to prevent cardiovascular episodes, neurodegenerative disease, macular degeneration and cancer. Little amounts of such lipophilic substances are necessary to carry out their antioxidant role, but the excessive intake may lead to unwanted redox imbalances which may provoke some disorders, and their use as complements is sometimes under debate.

Ascorbic acid (vitamin C) is synthesized in the majority of living beings, excepting primates (including humans), guinea pigs, bats and some birds. This makes our dependency of external vitamin C provision (basically plant products) strictly necessary. In animals and humans, vitamin C stimulates enzymes involved in the biosynthesis of collagen, catecholamines, L-carnitine, cholesterol, and amino acids, as well as in the hormonal activation, histamine detoxification, and phagocytosis by leukocytes, among others. In addition, vitamin C is linked to the reduction of incidence of a series of pathologies related to cancer, blood pressure and cardiovascular diseases prevention, immunity dysfunction, tissue regeneration, nervous system, etc. A lack of vitamin C causes scurvy, which leads to fragility of blood vessels and damage of connective tissue. Then, a failure in the collagen production takes place that may end in death as a consequence of the general collapse.

In plants, ascorbate is synthesized in mitochondria but its main target as powerful antioxidant is the chloroplast, where besides scavenging diverse ROS, as referred above, it is integrated in the AGC for hydrogen peroxide removal. Besides, ascorbate can regenerate α-tocopherol and participates in the xanthophylls’ cycle, one type of carotenoids. Thus, due to its functionality, ascorbate is necessary in high amounts (as α-tocopherol and carotenoids are), not only in chloroplasts but also in other plant organelles. An example of the dualism antioxidant/vitamin and regarding ascorbate is as follows: (i) one pepper fruit from the California type (about 250 g) contains 350–375 mg [1]; (ii) one pepper plant is able to yield about 10 Kg fruits, which means around 14 g vitamin C, only in fruits, without considering leaves and other organs (pepper is a factory for vitamin C production); (iii) however, our daily requirements are about 80 mg of this vitamin; (iv) thus, one such pepper plant can satisfy the daily requirements of about 175 people. Why such a difference? The common physiological conditions imposed by light in plants, from an antioxidant-consuming perspective, are much more stressful than those operating in the majority of our physiological dysfunctions and diseases.

Following this rationale about the different viewpoints depending on the target organism, either human, animal or plant, this Special Issue will cover the same question posed in the title and developed on above for antioxidant/vitamin concepts. Thus, the concept of antioxidants in the transition from the field to the body, either human or animal, is exemplified using antocyanins as the case study [2]. Several plant materials have been used as vehicle to provide antioxidants for our diet. Thus, pepper fruits have been shown as one of the most important ascorbate sources, although the global provision of vitamin C upon the pepper intake depends on the variety and the ripening stage of fruits [1,3]. Moreover, in some cases, this antioxidant provision is also complemented by some functional compounds which, in the case of pepper, includes capsaicin, an alkaloid exclusive of this species with diverse therapeutic properties [1]. Variety and ripening are also relevant cues in the antioxidant content (ascorbate, polyphenols, flavonoids) of other noteworthy fruits in our diet, such as the *Citrus* species (Mandarin, Kumquat and Clementine), apples and grapes, and this antioxidant capacity is extensive to any of their parts, either pulp, seeds and peel/skin [4,5,6]. Likewise, fruits are also sources of other important antioxidants, i.e., α-tocopherol (vitamin E) and tocochromanols. It was proved that avocados are very rich in these compounds, always depending on the variety and the storage conditions [7]. Plants are also used to brew antioxidant-enriched beverages like tea. The type of tea, either white, green, black and red has been found to be essential for the preparation of Kombucha, a beverage obtained from fermented tea [8].

Many plant products are used for the food industry, giving rise to a huge diversity of manufactured goods undergoing a series of processes, which do not necessarily imply transformation. This is the case for stevia, which is a sweetener directly extracted from the original plant without any transformation. In this Special Issue, it is reported that the antioxidant metabolism of stevia is influenced by the acclimation of plants when they are exposed to ex vitro conditions [9].

Virgin olive oil deserves a special mention as a particular source of antioxidants [10], greatly attributed to its high content in polyphenols. Although the phenolic profile of extra virgin olive oil (EVOO) is modified when cooking, EVOO maintains its nutritional parameters and properties within the EU’s health claims [11].

In the chapter of transformed products, pasta is one of the most paradigmatic foods obtained from wheat. Pasta can be used as a functional product since it is often consumed and it contains certain levels of phenolic compounds. Studies of bioaccesibility and bioavailability of such antioxidants can help formulating pasta where by-products could be even used as functional ingredients after the application of some pre-processing technologies [12].

Undoubtedly, all screening and quantification assays of antioxidants contained in natural or manufactured foods should be updated to gain accuracy, liability and reproducibility, so the nutritional standards can be fit through the use of consensual methods and approaches. Such a critical review of existing assays and a prospect analysis has been achieved in this Special Issue [13].

This volume also includes a series of articles focused on the role of food antioxidants in the prevention of chronic diseases and health disorders. Thus, the relevance of lycopene, a carotenoid which is abundant in tomato, has been highlighted in certain diseases including diabetes mellitus, cardiac complications, cancer insurgences, oxidative stress-mediated malfunctions, inflammatory, skin and bone diseases, as well as reproductive, hepatic and neural disorders [14]. It has been also reported that carotenoid derivatives inhibit osteoclast differentiation through the partial inhibition of the NF-ƙB pathway. Additionally, they can synergistically inhibit osteoclast differentiation as well in the presence of curcumin and carnosic acid [15].

This goodness of natural food for our health is in some cases undermined by processing. Thus, in the analysis of the antioxidant activity of green tea once it was processed, it was found that food processing does not always have positive effect on products destined for human consumption [16]. In this line, a gamma-conglutin found in narrow-leafed lupin showed anti-inflammatory properties through the promotion of insulin resistance and amelioration of the potential oxidative stress underwent in PANC-1 pancreatic cells [17]. Likewise, using Raw 264.7 murine, it was found that root cell extracts from curly dock (*Rumex crispus*) displayed anti-inflammatory and anticancer activities, possibly due to the anthraquinone present in this plant material [18]. In the same animal model, it was also demonstrated the antioxidant potential of elephant apple (*Dillenia indica* L.) bark against *tert*-butyl-hydroperoxide)-induced oxidative stress [19].

Strategies of (micro)encapsulation of materials and how they can contribute to benefit human health are another trend which is gaining attention nowadays. The use of encapsulating red cabbage extracts to improve the bioaccesibility of their polyphenols has been reported to be a good choice to counteract the diseases associated to oxidative stress episodes [20]. Similarly, it was reported that microencapsulation of Saskatoon Berry fruit (*Amelanchier alnifolia*) might be a promising practice with positive impact in the functionality of rye bread [21].

Research on antioxidant capacity of foods coming from animal sources has been also included in this Special Issue. By making comparisons of the meat origin, either conventional or organic, from selected elements of the pork carcass (ham, loin and shoulder), it was observed that meat products from conventional rearing systems had the best antioxidant properties with respect to antioxidant peptides. This discovery could be addressed to the human health field but also to the related industry [22].

Globally, this volume compiles a series of articles focused on the potential contribution of consuming certain food products for the human welfare. The basis of such benefits is mainly related to the antioxidant contribution of those products, but their content of other health promoting compounds cannot be discarded. This drives our attention to investigate those products and their chemical composition to convert them into functional foods with nutraceutical properties. Much research is necessary to understand the specific mechanisms by which many secondary plant metabolites act over our physiology in a beneficial manner and how they interact to prevent a number of human disorders.

## References

[1] Palma J.M., Terán F., Contreras-Ruiz A., Rodríguez-Ruiz M., Corpas F.J. (2020). Antioxidant profile of pepper (*Capsicum annuum* L.) fruits containing diverse levels of capsaicinoids. Antioxidants.

[2] Bendokas V., Stanys V., Mažeikienė I., Trumbeckaite S., Baniene R., Liobikas J. (2020). Anthocyanins: From the field to the antioxidants in the body. Antioxidants.

[3] Fratianni F., d’Acierno A., Cozzolino A., Spigno P., Riccardi R., Raimo F., Pane C., Zaccardelli M., Tranchida Lombardo V., Tucci M. (2020). Biochemical characterization of traditional varieties of sweet pepper (*Capsicum annuum* L.) of the Campania region, Southern Italy. Antioxidants.

[4] Nawel Benbouguerra N., Richard T., Saucier C., Garcia F. (2020). Voltammetric behavior, flavanol and anthocyanin contents, and antioxidant capacity of grape skins and seeds during ripening (*Vitis vinifera* var. Merlot, Tannat, and Syrah). Antioxidants.

[5] Costanzo G., Iesce M.R., Naviglio D., Ciaravolo M., Vitale E., Arena C. (2020). Comparative studies on different citrus cultivars: A revaluation of waste mandarin components. Antioxidants.

[6] Zielińska D., Turemko M. (2020). Electroactive phenolic contributors and antioxidant capacity of flesh and peel of 11 apple cultivars measured by Cyclic Voltammetry and HPLC-DAD5 MS/MS. Antioxidants.

[7] Vincent C., Mesa T., Munné-Bosch S. (2020). Identification of a new variety of avocados (*Persea americana* Mill. CV. Bacon) with high vitamin E and impact of cold storage on tocochromanols composition. Antioxidants.

[8] Jakubczyk K., Kałduńska J., Kochman J., Janda K. (2020). Chemical profile and antioxidant activity of the Kombucha beverage derived from white, green, black and red tea. Antioxidants.

[9] Acosta-Motos J.R., Noguera-Vera L., Barba-Espín G., Piqueras A., Hernández J.A. (2019). Antioxidant metabolism and chlorophyll fluorescence during the acclimatisation to ex vitro conditions of micropropagated *Stevia rebaudiana* bertoni plants. Antioxidants.

[10] Borges T.H., Serna A., López L.C., Lara L., Nieto R., Seiquer I. (2019). Composition and antioxidant properties of Spanish extra virgin olive oil regarding cultivar, harvest year and crop stage. Antioxidants.

[11] Lozano-Castellón J., Vallverdú-Queralt A., Rinaldi de Alvarenga J.F., Illán M., Torrado-Prat X., Lamuela-Raventós R.M. (2020). Domestic sautéing with EVOO: Change in the phenolic profile. Antioxidants.

[12] Melini V., Melini F., Acquistucci R. (2020). Phenolic compounds and bioaccessibility thereof in functional pasta. Antioxidants.

[13] Sadeer N.B., Montesano D., Albrizio S., Zengin G., Mahomoodally M.F. (2020). The versatility of antioxidant assays in food science and safety—Chemistry, applications, strengths, and limitations. Antioxidants.

[14] Imran M., Ghorat F., Ul-Haq I., Ur-Rehman H., Aslam F., Heydari M., Shariati M.A., Okuskhanova E., Yessimbekov Z., Thiruvengadam M. (2020). Lycopene as a natural antioxidant used to prevent human health disorders. Antioxidants.

[15] Odes-Barth S., Khanin M., Linnewiel-Hermoni K., Miller Y., Abramov K., Levy J., Sharoni Y. (2020). Inhibition of osteoclast differentiation by carotenoid derivatives through inhibition of the NF-ƙB pathway. Antioxidants.

[16] Xu X.Y., Zheng J., Meng J.M., Gan R.Y., Mao Q.Q., Shang A., Li B.Y., Wei X.L., Li H.B. (2019). Effects of food processing on in vivo antioxidant and hepatoprotective properties of green tea extracts. Antioxidants.

[17] Lima-Cabello E., Alché J.D., Morales-Santana S., Clemente A., Jimenez-Lopez J.C. (2020). Narrow-leafed lupin (*Lupinus angustifolius* L.) seeds gamma-conglutin is an anti-inflammatory protein promoting insulin resistance improvement and oxidative stress amelioration in PANC-1 pancreatic cell-line. Antioxidants.

[18] Eom T., Kim E., Kim J.S. (2020). In vitro antioxidant, antiinflammation, and anticancer activities and anthraquinone content from *Rumex crispus* root extract and fractions. Antioxidants.

[19] Alam M.B., Islam S., Ahmed A., Choi H.J., Motin M.D., Kim S., Lee S.H. (2020). Phytochemical characterization of *Dillenia indica* L. bark by paper spray ionization mass spectrometry and evaluation of its antioxidant potential against t-BHP-induced oxidative stress. Antioxidants.

[20] Izzo L., Rodríguez-Carrasco Y., Pacifico S., Castaldo L., Narváez A., Ritieni A. (2020). Colon bioaccessibility under in vitro gastrointestinal digestion of a red cabbage extract chemically profiled through UHPLC-Q-Orbitrap HRMS. Antioxidants.

[21] Lachowicz S., Świeca M., Pejcz E. (2020). Improvement of health-promoting functionality of rye bread by fortification with free and microencapsulated powders from *Amelanchier alnifolia* Nutt. Antioxidants.

[22] Kęska P., Rohn S., Halagarda M., Wójciak K.M. (2020). Peptides from different carcass elements of organic and conventional pork—Potential source of antioxidant activity. Antioxidants.

